# Women empowerment as an enabling factor of contraceptive use in sub-Saharan Africa: a multilevel analysis of cross-sectional surveys of 32 countries

**DOI:** 10.1186/s12978-018-0658-5

**Published:** 2018-12-20

**Authors:** Sanni Yaya, Olalekan A. Uthman, Michael Ekholuenetale, Ghose Bishwajit

**Affiliations:** 1 School of International Development and Global Studies, 120 University Private, University of Ottawa, Ottawa, ON K1N 6N5 Canada; 2 Warwick Centre for Applied Health Research and Delivery (WCAHRD), Division of Health Sciences, Warwick Medical School, University of Warwick, Coventry, CV4 7AL UK; 3 Department of Epidemiology and Medical Statistics, Faculty of Public Health, College of Medicine, University of Ibadan, Ibadan, Nigeria

**Keywords:** Women’s empowerment, Gender equality, Decision making, Sub-Saharan Africa, Antenatal care, Maternal health

## Abstract

**Background:**

Women’s empowerment has a direct impact on maternal and child health care service utilization. Large scope measurement of contraceptive use in several dimensions is paramount, considering the nature of empowerment processes as it relates to improvements in maternal health status. However, multicountry and multilevel analysis of the measurement of women’s empowerment indicators and their associations with contraceptive use is vital to make a substantial intervention in the Sub-Saharan Africa context. Therefore, we investigated the impact of women’s empowerment on contraceptive use among women in sub-Saharan Africa countries.

**Methods:**

Secondary data involving 474,622 women of reproductive age (15–49 years) from the current Demographic and Health Survey (DHS) in 32 Sub-Saharan Africa region was used in this study. Contraceptive use was the primary outcome variable. Multilevel analysis was conducted to examine the impact of women’s empowerment on contraceptive use. Percentages were conducted in univariate analysis. Furthermore, multilevel logistic regression models were used to analyze the association between individual, compositional and contextual factors of contraceptive use.

**Results:**

Results showed large disparities in the number of women who reportedly ever use contraceptive methods; this range from as low as 6.7% in Chad and as much as 72% in Namibia. More than one-third of the respondents had no formal education and more than half were active labor force. Contraceptive use was significantly more common among respondents from the richest households (28.5% versus 18.9%). Various components of women’s empowerment were positively significantly associated with contraceptive use after adjusting for demographic and socioeconomic factors. There was a significant variation in the odds of contraceptive use across the 32 countries (*σ*^2^= 1.12, 95% CrI 0.67 to 1.87) and across the neighbourhoods (*σ*^2^= 0.95, 95% CrI 0.92 to 0.98).

**Conclusions:**

Our findings suggest that an increase in contraceptive use and by better extension maternal health care services utilization can be achieved by enhancing women’s empowerment. Also, an increase in decision-making autonomy by women, their participation in labour force, reduction in abuse and violence and improved knowledge level are all key issues to be considered. Health-related policies should address inequalities in women’s empowerment, education and economic status which would yield benefits to individuals, families, and societies in general.

## Plain English summary

Approximately 99% of the global maternal mortality occurred in resource-constrained settings, with Sub-Saharan Africa region reporting about two-third (66%) of maternal deaths. Literature showed that women’s status within the household is a prominent factor for improving the utilization of maternal health services. Nevertheless, the impact of women’s socio-economic class on maternal health care use has not received adequate attention in Sub-Saharan Africa.

We have undertaken nationally representative Demographic and Health Surveys (DHS) data involving the selection of 32 countries based on geographical diversity. We used a 3-level model to explore contextual and compositional factors associated with contraceptive use. Neighbourhood socioeconomic disadvantage was operationalized with a principal component approach using key indicators. More so, Human Development Index (HDI) was used as a measure of a country’s intensity of deprivation, which was the average percentage of deprivation experienced by people in multidimensional poverty. Contextual effects were measured by the intraclass correlation (ICC) and median odds ratio (MOR). There were large disparities in contraceptive use, with about half of the study countries identified with low contraceptive use. This study empirically demonstrated the individual-level, neighbourhood-level and country-level maternal factors associated with contraceptive use among women in Sub-Saharan African countries.

Based on the findings of contraceptive use and its associated women’s empowerment and proximate factors among women of reproductive age in Sub-Saharan Africa countries, there is a need for regional interventions in improving fertility control measures, specifically contraceptive use.

## Introduction

The Sustainable Development Goal (SDG-5) targets to achieve gender equality and empowerment of all women and girls [1]. Reducing gender inequality is a vital policy agenda globally, through access and rights to resources. To enhance the impacts of programs and policies that relate to gender, a crucial step is first to understand how inequality is exhibited throughout the world, specifically, by identifying where it occurs using indicators and approaches that are consistently disaggregated as to find a resolution as possible. Such clear measure of indicators for gender inequality is not known to currently exist. In the past decades, assessments of gender disparity in many parts of the world have failed to deliver on promoting gender equality and empower women [2–4], with evidence suggesting that women in some countries have seen decreased opportunities to improve their welfare [5]. One reason gender equality is so high on the international policy concern is the growing body of evidence showing that improving the welfare of women and closing the inequality gap can lead to improved childhood nutrition and reduced mortality, increased school enrollment, improved maternal and children’s health and improved natural resource management [6].

Maternal autonomy in healthcare-seeking behaviour is connected to women’s empowerment and helps to achieve desired health outcomes [7]. The multifaceted latent nature of women’s empowerment makes it difficult for researchers to directly and accurately measure empowerment. Generally, proxy indicators are commonly used to measure empowerment, including but not limited to decision making power, reasons to justify sexual violence, women’s knowledge level, and labour force participation [8]. In most communities, specifically in Sub-Saharan Africa region, men have huge control over the women of their social class; particularly within families and households [6]. The health care system for children and mothers alike in many communities is poorly affected by the women’s subservient status within households, which is consequent upon social and cultural predetermined roles for women that subvert almost every aspect of their lives [9]. Thus, women’s empowerment is majorly recognized as a vital tool to enable access to reproductive and sexual health care services for improved mother-and-child health [10].

Debate related to the prominent indicators for measuring women’s empowerment has established that women’s empowerment can be evaluated by their capability to contribute in household decision-making which reflects their economic, domestic and movement autonomies [7, 11]. The more empowered women are, the more likely to use modern contraception, deliver in a health facility and have a skilled attendant at birth [12]. In addition, contraceptive use is important in preventing fetal, neonatal, and under-5 deaths; reducing maternal mortality and avoiding high-risk pregnancy including pregnancy among teenage girls and older women. Many women with issues of health care challenges experience gendered power inequalities, especially in their intimate relationships, that prevent them from achieving optimal sexual and reproductive health benefits and exercising their rights [13]. An increasing body of evidence demonstrates the ways unequal levels of power between men and women in intimate relationships prevent women from making decisions regarding their sexual and reproductive health [14]. Frequently, unequal control over and access to economic resources, unequal relationship power, and limited ability to make sexual decisions (including whether, when, how often, and with whom to have sex; and negotiating condom use, contraceptive or other protective practices) make women vulnerable to SRH risks [15].

Empowerment is one such characteristic that may influence a woman’s experience of pregnancy, delivery, and postnatal care. Women’s empowerment leads to significant positive changes in many domains. Studies have found an association between increased empowerment and reduced mortality and morbidity [16, 17]. In terms of reproductive health, empowerment has been associated with reduced rates of unintended pregnancies [18] and sexually transmitted diseases, such as chlamydia and gonorrhea in high-risk populations [19]. Other reports show the benefits of empowerment for health-related behaviours such as obtaining nutritional supplements and participating in health education sessions [20]. The benefits of empowerment are not necessarily limited to women themselves but have the potential to extend to those around her, including but not limited most prominently to her own children.

Transformative strategies between gender helps to address gender inequalities while promoting health. These techniques support awareness of gender roles and intervene in the distribution of resources and allocation of responsibilities between male and female, handle power relationships and promote the position of women [17]. Women’s empowerment should be regarded as a crucial element and an indicator of maternal health. In this study, we investigated whether empowerment could help women utilize contraceptive services.

## Methods

### Data source

This study was conducted using individual woman component of Demographic and Health Survey (DHS). The study included 474,622 women of reproductive age (15–49 years). The data is publicly available and can be accessed from MEASURE DHS database at http://dhsprogram.com/data/available-datasets.cfm. This study used data from 32 recent DHS surveys conducted between 2008 and 2016 in Sub-Saharan Africa (SSA) available as of August 2018 and that included questions on contraceptive use among women [21]. The central Sub-Sahara countries include; Angola, Congo, Gabon and the Democratic Republic of Congo. The Eastern Sub-Saharan countries include; Burundi, Comoros, Ethiopia, Kenya, Malawi, Mozambique, Rwanda, Tanzania, Uganda, and Zambia. The Southern Sub-Saharan Africa countries include; Lesotho, Namibia, and Zimbabwe. The Western countries were; Nigeria, Guinea, Niger, Benin, Cameroon, Chad, Ghana, Burkina-Faso, Cote d’Ivoire, Liberia, Mali, Senegal, Sierra Leone, Togo and The Gambia. See the details of the countries in Table 1.

**Table 1:** Description of Demographic and Health Surveys data by countries, in sub-Saharan Africa, 2007 to 2016

					Human Development Index (HDI)	
Country	Survey year	Number of children	Number of neighbourhoods	Contraceptive (%)	Value	Category
Angola	2016	14,379	625	19.1	0.532	High HDI
Benin	2012	16,599	750	27.4	0.485	Moderate HDI
Burkina Faso	2010	17,087	573	24.1	0.402	Low HDI
Burundi^a^	2011	9389	376	20.8	0.404	Low HDI
Cameroon	2011	15,426	578	43.7	0.518	Moderate HDI
Chad^a^	2015	17,719	624	6.7	0.396	Low HDI
Comoros	2012	5329	252	22.5	0.727	High HDI
Congo	2012	10,819	384	69.3	0.592	High HDI
Congo DR^a^	2014	18,827	536	30.1	0.435	Low HDI
Cote d’Ivoire	2012	10,060	351	35.5	0.474	Moderate HDI
Ethiopia	2008	15,683	643	37.5	0.448	Low HDI
Gabon	2012	8422	336	49.6	0.697	High HDI
Gambia	2013	10,233	281	14.4	0.452	Low HDI
Ghana	2014	9396	427	42.7	0.579	High HDI
Guinea	2012	9142	300	15.2	0.414	Low HDI
Kenya	2014	31,079	1593	56.3	0.555	High HDI
Lesotho	2014	6621	399	69.6	0.497	Moderate HDI
Liberia^a^	2013	9239	322	34.5	0.427	Low HDI
Malawi	2016	24,562	850	68.7	0.476	Moderate HDI
Mali^a^	2013	10,424	413	20.7	0.442	Low HDI
Mozambique	2011	13,745	610	32.1	0.418	Low HDI
Namibia	2013	10,018	549	71.7	0.640	High HDI
Niger	2012	11,160	476	28.7	0.353	Low HDI
Nigeria	2013	38,948	896	24.2	0.527	Moderate HDI
Rwanda^a^	2015	13,497	492	45.7	0.498	Moderate HDI
Senegal	2015	8851	214	27.9	0.494	Moderate HDI
Sierra Leone^a^	2013	16,658	435	33.3	0.420	Low HDI
Tanzania	2016	13,266	608	48.7	0.531	High HDI
Togo	2014	9480	330	32.9	0.487	Moderate HDI
Uganda	2011	8674	404	44.3	0.493	Moderate HDI
Zambia	2014	16,411	721	54.9	0.579	High HDI
Zimbabwe	2015	9955	400	68.5	0.516	Moderate HDI

^a^Post-conflict countries

### Fertility rates in sub-Saharan Africa settings

Africa continent consists of fifty-four (54) sovereign nations, while six are in Northern Africa and the balance in SSA. Fertility rates differ substantially across SSA’s regions [22]. The total fertility rates which were about 6.5 births per woman in 1960s across all regions, currently range between 2.4 in Southern Africa to 3.1 in Northern Africa, 4.5 in Eastern Africa, and 5.2–5.3 in Western and Middle Africa [23]. The choice of large family size remains a major factor determining levels of fertility in SSA. Recent data from DHS brought to limelight the reasons why people prefer and choose to have large families [24]. Though the factors prompting women’s decisions are complex and differ from one location to the other, there exist some similarities. More so, religious beliefs, culture, low child survival rates and gender relations, including having male children in the family seem to play a major role in the decisions about reproduction and hence overall fertility levels and trends in SSA.

Developments in the health system have stimulated greater policy support for improving sexual and reproductive health (SRH), both directly and indirectly. International support for family planning improved substantially after the 2012 London Summit, co-sponsored by the Bill and Melinda Gates Foundation and the UK Government, in partnership with the United Nations Population Fund, national governments, other donors, civil society, and agencies from other sectors. The Summit issued a call for global and national commitments to enable 120 million more women and girls to use modern contraceptives by 2020 [25]. This has prompted 49 governments, including 39 from Africa and 10 others from the poorest 69 countries to make commitments towards achieving this goal. Also, the Summit has motivated governmental and non-governmental organizations to increase contraceptive supply and access through improved commodity supply chains and service delivery models; increased demand and support for family planning. Interestingly, a series of international family planning conferences, three of four having been held in the SSA region, appears to have provided added momentum to the London Summit [26].

### Variables measurement

#### Outcome

Contraceptive use was the primary outcome variable of this study. The lifetime contraceptive use of women was self-reported and ascertained from the question, “Ever used anything or tried to delay or avoid getting pregnant?”, with the response options “yes, used outside calendar”, “yes, used in calendar” or “no”. Women were classified as “contraceptive users” if the response was “yes, used outside calendar” or “yes, used in calendar” to contraceptive use and “non-contraceptive user” if the response was “no” to contraceptive use.

#### Individual-level factors

Women’s empowerment was used as the main focal predictor in this study. The components of women’s empowerment included: 1) Labour force participation (current employment status), 2) Disagreement with reasons to justify wife beating (reasons such as; burning food, neglect of children, refusal to have sex with husband, visitations without permission from husband and argument with husband). 3) Decision-making power (measured by visiting family members, respondent’s health care, house earning and household purchases) 4) Knowledge level (education level, read the newspaper, listen to the radio and watch television). This is consistent with the methods by previous authors [8]. Other explanatory variables included: current age (years) of a respondent (15–19, 20–24, 25–29, 30–34. 35–39, 40–44, 45-49 years), place of residence (urban vs rural), sex of household head (male vs female), wealth index (poorest, poorer, middle, richer, richest), age at first birth, number of children ever born, and husband’s education.

#### Neighbourhood-level factors

We used the term neighbourhood to describe clustering within the same geographical living environment. Neighbourhoods where based on sharing a common primary sample unit within the DHS data. The sampling frame for identifying the primary sample unit in the DHS is usually the most recent census. The unit of analysis was chosen for two reasons. First, the primary sample unit is the most consistent measure of neighbourhood across all the surveys [27], and thus the most appropriate identifier of neighbourhood for this cross-region comparison. Second, for most of the DHS conducted, the sample size per cluster meet the optimum size with a tolerable precision loss [27]. Neighbourhood socioeconomic disadvantage was operationalized with a principal component comprised of the proportion of respondents with no formal education, unemployed, rural resident, and living below the poverty level (asset index below 20% poorest quintile). A standardized score with mean 0 and standard deviation 1 was generated from this index; with higher scores indicative of the lower socioeconomic position (SEP). We divided the resultants scores into tertiles to allow for nonlinear effects and provide results that were more readily interpretable in the policy arena.

#### Country-level factor

Country-level data were collected from the reports published by the United Nations Development Program [28]. At country-level, we included the human development index, a measure of a country’s intensity of deprivation, which is the average percentage of deprivation experienced by people in multidimensional poverty. The Human Development Index (HDI) is a summary measure of average achievement in key dimensions of human development: a long and healthy life, being knowledgeable and have a decent standard of living. The HDI is the geometric mean of normalized indices for each of the three dimensions. The health dimension is assessed by life expectancy at birth, the education dimension is measured by mean of years of schooling for adults aged 25 years and more and expected years of schooling for children of school entering the age. The standard of living dimension is measured by gross national income (GNI) per capita. The HDI uses the logarithm of income, to reflect the diminishing importance of income with increasing GNI. The scores for the three HDI dimension indices are then aggregated into a composite index using geometric mean. The country-level variables were also categorized into three tertiles (low, middle and high levels). In addition, we included a country-level variable, ‘Post-Conflict country’, whether the country recently experiences conflict.

#### Control variable

The year the DHS was conducted was included as a partial control for a period trend to control for the effects of unknown factors that may have been introduced due to different timing of surveys across countries.

### Statistical analyses

#### Descriptive analyses

Data representation was adjusted for in all analyses to account for clustering, stratification and sample weight. In the descriptive statistics, the distribution of respondents by key variables were expressed as percentages.

#### Modeling approaches

**Fig. 1:**
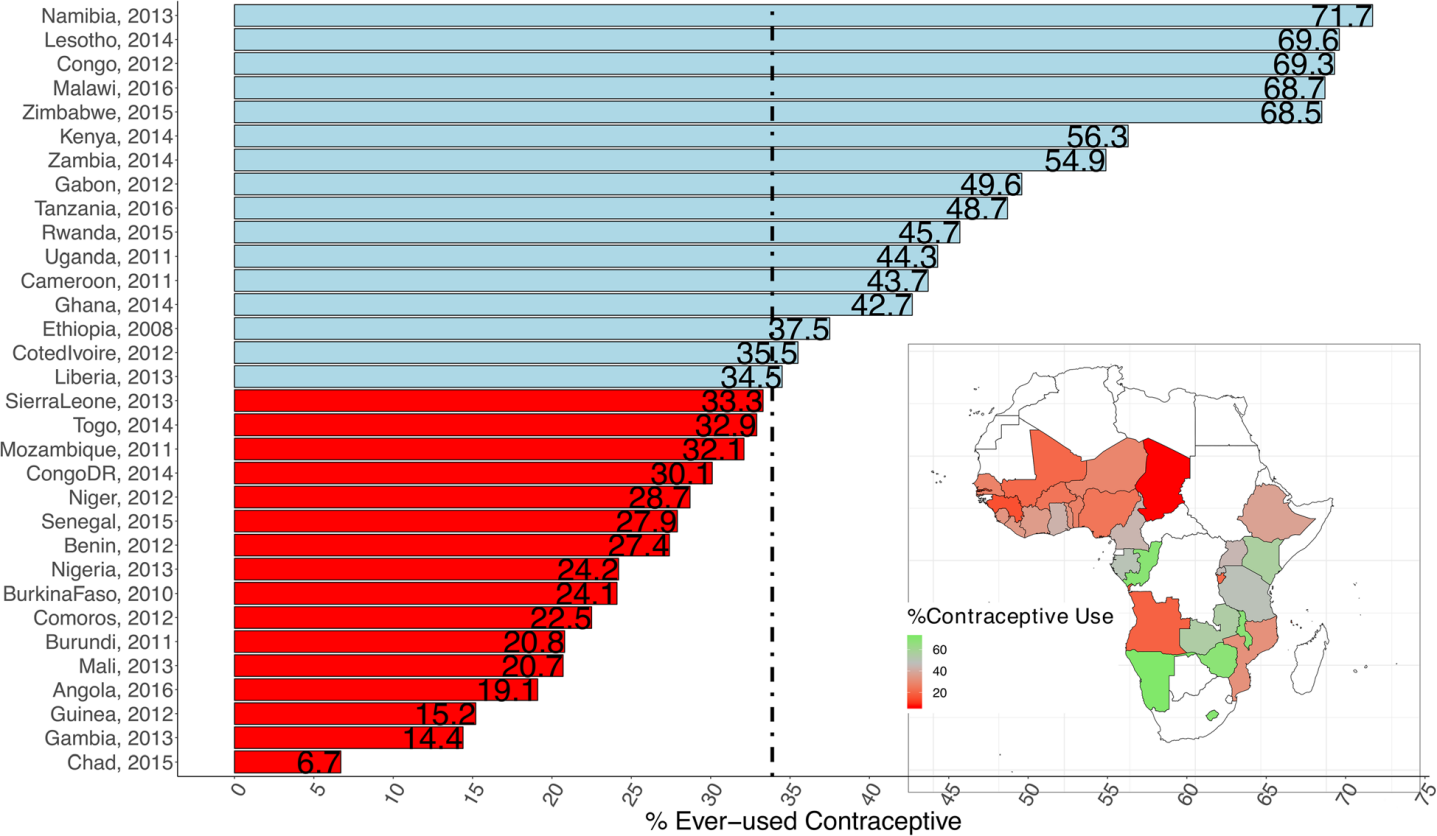
Percentage ever used contraceptive. Note: Red colouration implies below median value (33.88); light blue colouration implies above median value (33.88).

We used multivariable multilevel logistic regression models to analyze the association between individual, compositional and contextual factors associated with contraceptive use. We specified a 3-level model for binary response reporting contraceptive use or not, for women (at level 1), in a neighbourhood (at level 2) living in a country (at level 3) (see Fig. 1). We constructed five models. The first model, an empty or unconditional model without any explanatory variables, was specified to decompose the amount of variance that existed between country and neighbourhood levels. The second model contained only individual-level factors, the third model contained only neighbourhood-level factors, and the fourth model contained only country-level factors. Finally, the fifth model simultaneously controlled for individual, neighbourhood and country level factors (Full model).

#### Fixed effects (measures of association)

The results of fixed effects (measures of association) were reported as odds ratios (ORs) with their 95% credible intervals (CrIs). Bayesian statistical inference provides probability distributions for measures of association (ORs), which can be summarized with 95% credible intervals (95% CrI), rather than 95% confidence intervals (95% CI). A 95% credible interval can be interpreted as there is a 95% probability that the parameter takes a value in the specified range [29].

#### Random effects (measures of variation)

The possible contextual effects were measured by the intraclass correlation (ICC) and median odds ratio (MOR) [30, 31]. We measured the similarity between respondents in the same neighbourhood and within the same country using ICC. The ICC represents the percentage of the total variance in the probability of contraceptive use that is related to the neighbourhood and country level, i.e. measure of clustering of odds of contraceptive use in the same neighbourhood and country. The MOR measures the second or third level (neighbourhood or country) variance as odds ratio and estimates the probability of contraceptive use that can be attributed to neighbourhood and country context. MOR equal to one indicates no neighbourhood or country variance. Conversely, the higher the MOR, the more important are the contextual effects for understanding the probability of contraceptive use.

#### Model fit and specifications

We checked for multicollinearity among explanatory variables examining the variance inflation factor (VIF) [32], all diagonal elements in the variance-covariance (τ) matrix for correlation between − 1 and 1, and diagonal elements for any elements close to zero. None of the results of the tests provided reasons for concern. Thus, the models provide robust and valid results. The MLwinN software, version 3.01, was used for the analyses [33, 34]. Parameters were estimated using the Markov Chain Monte Carlo procedure [26]. The Bayesian Deviance Information Criterion was used as a measure of how well our different models fitted the data. A lower value on Deviance Information Criterion indicates a better fit of the model [35].

### Ethical approval

This study utilized secondary datasets. DHS Program is consistent with the standards for ensuring the protection of respondents’ privacy. ICF International ensures that the survey complies with the U.S. Department of Health and Human Services regulations for the respect of human subjects. No further approval was required for this study since the data is secondary and is available in the public domain. More details about data and ethical standards are available at http://goo.gl/ny8T6X.

## Results

### Sample characteristics

We analyzed information on 474,622 respondents (Level 1) nested within 16,748 neighbourhoods (Level 2) from 32 countries (Level 3) in Sub-Saharan Africa (Table 1). Table 1 shows the countries, year of data collection, and the surveys characteristics. The median number of neighbourhoods sampled was 455, ranging from 214 in Senegal to 1593 in Kenya. The median number of respondents was 10,990 (range: 5329 to 39,948). Seven countries could be classified as post-conflict countries. As shown in Fig. 1, there was a wide variation in the percentage of women who reported ever used contraceptive in the 32 countries studies. It ranged from as low as 6.7% in Chad to as much as 72% in Namibia. The characteristics of the pooled sample are shown in Table 2. Almost 40% of the respondents were aged between 15 to 24 years. More than one-third of the respondents had no formal education and more than half were in labor force. Contraceptive use was significantly more common among respondents from the richest households (28.5% versus 18.9%). In addition, women with partners with secondary or higher education reported more contraceptive use (48.6% versus 27.1%).

**Table 2:** Summary of pooled sample characteristics of the Demographic and Health Surveys data in sub-Saharan Africa

		Contraceptive use		
	Overall	No	Yes	*p*
	441,098	272,705	167,547	
Age (%)				< 0.001
15–24	174,203 (39.6)	127,692 (46.8)	46,510 (27.8)	
25–34	140,294 (31.9)	73,701 (27.0)	66,592 (39.7)	
35–49	125,759 (28.6)	71,312 (26.1)	54,445 (32.5)	
No of children (mean (sd))	2.87 (2.75)	2.65 (2.89)	3.23 (2.46)	< 0.001
Wealth (%)				< 0.001
Poorest	87,727 (19.9)	63,640 (23.3)	23,908 (14.3)	
Poorer	82,941 (18.8)	54,940 (20.1)	27,839 (16.6)	
Middle	83,487 (18.9)	52,480 (19.2)	30,861 (18.4)	
Richer	87,309 (19.8)	50,005 (18.3)	37,112 (22.2)	
Richest	99,634 (22.6)	51,640 (18.9)	47,827 (28.5)	
Partner’s education (%)				< 0.001
No education	107,685 (36.6)	85,067 (49.9)	22,419 (18.2)	
Primary	80,264 (27.3)	39,234 (23.0)	40,815 (33.2)	
Secondary+	106,313 (36.1)	46,298 (27.1)	59,774 (48.6)	
Labour force participation (yes, %)	243,911 (55.3)	140,671 (51.6)	102,918 (61.4)	< 0.001
Acceptance of wife beating (%)				< 0.001
Low	249,706 (56.6)	147,288 (54.0)	101,795 (60.8)	
Medium	68,756 (15.6)	45,831 (16.8)	22,924 (13.7)	
High	122,636 (27.8)	79,586 (29.2)	42,828 (25.6)	
Women’s knowledge level (%)				< 0.001
Low	197,536 (44.8)	135,993 (49.9)	61,043 (36.4)	
Medium	131,254 (29.8)	76,920 (28.2)	53,989 (32.2)	
High	112,308 (25.5)	59,792 (21.9)	52,515 (31.3)	
Decision making power				< 0.001
Low	47,485 (37.3)	27,943 (40.8)	19,485 (33.3)	
Medium	56,469 (44.4)	26,870 (39.3)	29,515 (50.4)	
High	23,210 (18.3)	13,642 (19.9)	9568 (16.3)	
Neighbourhood SES (%)				< 0.001
Tertile 1 (least disadvantaged)	148,852 (33.7)	78,012 (28.6)	70,635 (42.2)	
Tertile 2	145,590 (33.0)	88,626 (32.5)	56,680 (33.8)	
Tertile 3 (most disadvantaged)	146,656 (33.2)	106,067 (38.9)	40,232 (24.0)	
Human Development Index (%)				< 0.001
Low HDI	159,306 (36.1)	119,123 (43.7)	40,183 (24.0)	
Moderate HDI	162,673 (36.9)	94,496 (34.7)	68,173 (40.7)	
High HDI	119,119 (27.0)	59,086 (21.7)	59,191 (35.3)	

### Measures of associations (fixed effects)

The results of the different models are shown in Table 3. In the fully adjusted model controlling for the effects of individual, neighbourhood and country level factors, all the factors remained significantly associated with odds of contraceptive use.

**Table 3:** Individual compositional and contextual factors associated with contraceptive use in sub-Saharan Africa identified by multivariable multilevel logistic regression models, Demographic and Health Surveys data

	Model 1^a^	Model 2^b^	Model 3^c^	Model 4^d^	Model 5^e^
	OR (95% CrI)	OR (95% CrI)	OR (95% CrI)	OR (95% CrI)	OR (95% CrI)
Fixed-effect					
Control variable					
Survey years					
2008		1 (reference)	1 (reference)	1 (reference)	1 (reference)
2010		0.07 (0.04 to 0.12)	0.43 (0.20 to 0.76)	0.86 (0.44 to 1.91)	0.22 (0.07 to 0.62)
2011		0.37 (0.12 to 0.67)	1.00 (0.76 to 1.25)	0.52 (0.37 to 0.65)	0.28 (0.16 to 0.39)
2012		0.23 (0.16 to 0.33)	0.85 (0.58 to 1.19)	0.36 (0.29 to 0.43)	0.31 (0.14 to 0.52)
2013		0.20 (0.11 to 0.39)	0.73 (0.51 to 0.94)	0.62 (0.53 to 0.76)	0.18 (0.11 to 0.31)
2014		0.48 (0.31 to 0.74)	1.86 (1.55 to 2.16)	0.83 (0.69 to 1.00)	1.14 (0.68 to 1.85)
2015		0.79 (0.11 to 2.04)	0.74 (0.53 to 1.10)	0.77 (0.52 to 1.05)	0.20 (0.07 to 0.37)
2016		0.66 (0.17 to 1.01)	1.38 (0.92 to 1.95)	0.34 (0.19 to 0.53)	1.68 (0.36 to 4.21)
Individual-level factors					
Age (%)					
15–24		1 (reference)			1 (reference)
25–34		1.21 (1.16 to 1.27)			1.19 (1.12 to 1.24)
35–49		0.78 (0.74 to 0.82)			0.75 (0.71 to 0.79)
No of children		1.16 (1.14 to 1.17)			
Wealth (%)					
Poorest		1(reference)			1 (reference)
Poorer		1.37 (1.30 to 1.45)			1.24 (1.17 to 1.31)
Middle		1.62 (1.53 to 1.72)			1.33 (1.26 to 1.41)
Richer		2.17 (2.04 to 2.32)			1.60 (1.50 to 1.71)
Richest		2.93 (2.73 to 3.15)			1.98 (1.82 to 2.13)
Partner’s education (%)					
No education		1 (reference)			1 (reference)
Primary		1.69 (1.61 to 1.77)			1.62 (1.55 to 1.69)
Secondary+		2.02 (1.93 to 2.12)			1.91 (1.83 to 2.00)
Labour force participation (yes, %)		1.14 (1.07 to 1.22)			1.14 (1.06 to 1.21)
Acceptance of wife beating (%)					
Low		1 (reference)			1 (reference)
Medium		1.13 (1.08 to 1.19)			1.13 (1.08 to 1.18)
High		0.98 (0.96 to 1.04)			1.01 (0.97 to 1.04)
Women’s knowledge level (%)					
Low		1 (reference)			1 (reference)
Medium		1.51 (1.45 to 1.57)			1.46 (1.40 to 1.52)
High		2.04 (1.94 to 2.14)			1.96 (1.87 to 2.05)
Decision making power					
Low		1 (reference)			1 (reference)
Medium		1.23 (1.19 to 1.27)			1.21 (1.16 to 1.26)
High		1.25 (1.20 to 1.31)			1.23 (1.18 to 1.29)
Neighbourhood factor					
Neighbourhood SES (%)					
Tertile 1 (least disadvantaged)			1 (reference)		1 (reference)
Tertile 2			0.65 (0.63 to 0.68)		0.76 (0.71 to 0.81)
Tertile 3 (most disadvantaged)			0.31 (0.29 to 0.32)		0.43 (0.40 to 0.46)
Country-level factor					
Conflict (yes vs no)				0.82 (0.66 to1.05)	1.20 (0.80 to 1.91)
Human Development Index (%)					
Low HDI				1 (reference)	1 (reference)
Moderate HDI				2.83 (2.59 to 3.19)	1.56 (1.22 to 2.47)
High HDI				3.75 (3.21 to 4.32)	1.75 (0.95 to 2.68)
Random-effect					
Country-level					
Variance (95% CrI)	1.12 (0.67 to 1.87)	1.85 (1.06 to 3.17)	1.00 (0.60 to 1.69)	0.74 (0.44 to 1.23)	1.90 (0.99 to 3.47)
VPC (%, 95 CrI)	20.9 (13.7 to 30.4)	30.5 (20.3 to 42.6)	19.9 (13.1 to 29.5)	31.2 (19.2 to 45.0)	31.2 (19.2 to 45.0)
MOR (95% CrI)	2.75 (2.18 to 3.68)	3.66 (2.67 to 5.46)	2.59 (2.09 to 3.45)	3.73 (2.58 to 5.91)	3.73 (2.58 to 5.91)
Neighbourhood-level					
Variance (95% CrI)	0.95 (0.92 to 0.98)	0.93 (0.87 to 0.98)	0.72 (0.70 to 0.74)	0.95 (0.92 to 0.98)	0.90 (0.85 to 0.94)
VPC (%, 95 CrI)	38.6 (32.6 to 46.4)	45.8 (37.0 to 55.8)	34.3 (28.2 to 42.5)	33.9 (29.2 to 40.1)	46.0 (35.8 to 57.3)
MOR (95% CrI)	2.53 (2.50 to 2.57)	2.51 (2.43 to 2.57)	2.25 (2.22 to 2.28)	2.53 (2.50 to 2.57)	2.47 (2.41 to 2.53)
Model fit statistics					
DIC	474,622	122,624	473,685	474,632	122,165
Sample size					
Country-level	32	32	32	32	32
Neighbourhood-level	16,748	15,344	16,748	16,748	15,344
Individual-level	440,052	123,258	440,052	440,052	123,258

^a^Model 1 – empty null model, baseline model without any explanatory variables (unconditional model)

^b^Model 2 – adjusted for only individual-level factors

^c^Model 3 – adjusted for only neighbourhood-level factors

^d^Model 4 – adjusted for only country-level factors

^e^Model 5 – adjusted for individual-, neighbourhood-, and country-level factors (full model)

*OR* odds ratio, *CrI* credible interval, *MOR* median odds ratio, *VPC* variance partition coefficient, *DIC* Bayesian Deviance Information Criteria

Women aged 25 to 34 years old were more likely to have used contraceptive compared to those aged 15 to 24 years old (OR = 1.19, 95% CrI 1.12 to 1.24). Women from the richest households were as twice as likely to have used contraceptive than those from poorest households (OR = 1.98, 95% CrI 1.82 to 2.13). Women whose partner had secondary or higher were also almost as twice as likely to have used contraceptive than those whose partner had no education (OR = 1.81, 95% CrI 1.83 to 2.00). Women in labour were 14% more likely to have used contraceptive than those not working (OR = 1.14, 95% CrI 1.06 to 1.21). Women with medium acceptance of wife beating were 13% more likely to have used contraceptive than those with low acceptance of wife beating (OR = 1.13, 95% CrI 1.08 to 1.18). Women with high knowledge level were as twice as likely to have used contraceptive than those with low knowledge level (OR = 1.96, 95% CrI 1.87 to 2.05). Women with the high decision-making power were 23% more likely to have used contraceptive than those with the low decision-making power (OR = 1.23, 95% CrI 1.18 to 1.29).

Women living in the most SEP disadvantaged neighbourhood were 57% less likely to have used contraceptive than those in the least SEP disadvantaged neighbourhood (OR = 0.43, 95% CrI 0.40 to 0.46). Women from countries with moderate human development index were 1.56 times more likely to have used contraceptive than those from countries with low human development index (OR = 1.56, 95% CrI 1.22 to 2.47).

### Measures of variations (random effects)

As shown in Table 3, in Model 1 (unconditional model), there were substantial variations in the odds of contraceptive use across the 32 countries (*σ*^2^= 1.12, 95% CrI 0.67 to 1.87) and across the neighbourhoods (*σ*^2^= 0.95, 95% CrI 0.92 to 0.98). Having 20.9 and 38.6% VPC estimates for the model implied that the variance in odds of contraceptive use could be attributed to country and neighbourhood level factors, respectively. Results from the median odds ratio (MOR) also confirmed evidence of neighbourhood and societal contextual phenomena shaping contraceptive use. From the full model (Model 5), it was estimated that if a women moved to another country or neighbourhood with a higher probability of contraceptive use, the median increase in their odds of contraceptive would be 3.73 (95% CrI 2.58 to 5.91) and 2.47-fold (95% CrI 2.41 to 2.53) respectively.

## Discussion

The results of this study showed large disparities in contraceptive use across Sub-Saharan Africa countries. The findings also revealed low contraceptive use from several countries including Chad, Gambia, Guinea, Angola, Mali, Burundi, Comoros, Burkina-Faso and Nigeria where less than one-quarter of the women had ever used a contraceptive method. Similarly, in recent studies, there were reports of low contraceptive use in developing countries [18, 36–38]. Here, our study also investigated the association between contraceptive use and various components of women’s empowerment. The significant association between women’s empowerment and contraceptive use is consistent with the results from previous studies where women’s empowerment was positively associated with the use of health care services in 67 developing countries [38]. While several studies from individual countries in Africa have consistently shown the demographic and socio-economic factors associated with contraceptive use, this study employed a multi-country approach in Sub-Saharan Africa region to establish the association between women’s empowerment and contraceptive use.

The pooled multicountry analyses accounted for the heterogeneity across different levels of factors included in the model. We extensively examined women’s empowerment as a strong factor in contraceptive utilization, considering individual-level, neighbourhood-level, and country-level evidence. Women with higher knowledge level, those who participated in the labour force, with higher wealth status, and having more decision-making power were found to have increased in the odds of contraceptive use. These findings are consistent to previous studies which identified maternal socioeconomic status such as education and wealth index as significantly associated with service utilization [36, 38, 39]. Partner’s education was also associated with improved contraceptive use. In terms of improved decision-making autonomy, access to economic resources, enhanced knowledge, low disadvantaged neighbourhood and countries with high human development index, such women would have greater chances to effectively cope with issues presented by socio-cultural, religious and health systems factors. The could be the basis for underscoring the findings of this study. High rates of maternal and child mortalities can be prevented through lower fertility rates and prolonged birth spacing, which involves contraceptive use through enhanced participation of women in labour force, protection against abuse and violence, higher knowledge, improved decision making power and socioeconomic status.

This study builds on the vast literature based on DHS data to describe components of women’s empowerment by considering standard indicators of women’s empowerment across Sub-Saharan Africa countries [40, 41]. The validated measure becomes a crucial instrument for global health researchers in determining the influence of women’s empowerment on contraceptive use and other health outcomes. In the same vein, the measure allows for comparisons across various countries in Sub-Saharan Africa while accounting for unique features of each context. Therefore, concerted efforts are paramount to improve women’s economic empowerment and promote business enterprises. Particularly, local savings groups, community and microfinance banks will aid African women who may not have surety for loans from financial institutions [42]. Also, the scaling up of lucrative medium and small-scale businesses should be supported by favourable trade policies and financial institutions. Promoting women’s economic empowerment through local support groups, charitable organizations or well-off individuals, trade ventures and effective policies would result in increased empowerment [43, 44].

### Strengths and limitations

The major strength of this study is the use of current nationally representative datasets from 32 different Sub-Saharan Africa countries, which makes the findings of the study generalizable to women of reproductive age in Sub-Saharan Africa countries. However, this analysis has some drawbacks. Prominently, the analyses utilized cross-sectional data, hence, only associations and no causal relationships are established. More so, our inability to measure sources of demand-side unobserved heterogeneity across the secondary data might have biased our estimates of correlates of contraceptive use. The unavailability of relevant variables was a major limitation in the DHS data. Hence, we considered supply-side limitations of data to basically those issues related to health care delivery. DHS did not report availability, accessibility, and frequency of utilization of contraceptive methods. Furthermore, recall bias could have occurred in this study that deals with life time contraceptive use.

## Conclusion

Based on the findings of this study, it is imperative to note that enhancing contraceptive use among women could help to ameliorate their health care services. This study showed that contraceptive use is associated with women’s empowerment and other proximate determinants; including partner’s education, maternal age, wealth or socioeconomic status, individual-level, neighbourhood-level and country-level factors. Improving women’s participation in labour force, which implies creating employment opportunities, reduction in gender-based violence, enhancing the decision making the power of women and increasing their knowledge level can improve contraceptive use and therefore achieve better maternal health in Sub-Saharan Africa countries. Furthermore, the development of community-based women’s empowerment programmes, such as women’s access to media and health information could be useful interventions to empower women. The subject of women’s empowerment on maternal health care could consider various pathways from empowerment to action such as maternal autonomy to sexual and reproductive health care including family planning services to expedite achievement of the fifth sustainable development goal. However, further policy research aimed at evolving plans and strategies to promote widespread contraceptive utilization as an integral part of an overall programme to improve efficiency need to focus on two key areas; availability and accessibility issues. In addition, future research should explore reasons for low prevalence in contraceptive use using qualitative approach or should consider prospective studies using panel data, asking respondent and partners about their contraceptive use, or repeating questions in different ways to check the consistency of the answers to solve the problem of recall bias that is inherent in this study.

## Abbreviations

DHS: Demographic health survey
SDGs: Sustainable development goals
SEP: Socio-economic position
SRH: Sexual and reproductive health
